# Graphene Oxide Nanosheets Interact and Interfere with SARS‐CoV‐2 Surface Proteins and Cell Receptors to Inhibit Infectivity

**DOI:** 10.1002/smll.202101483

**Published:** 2021-05-14

**Authors:** Mehmet Altay Unal, Fatma Bayrakdar, Hasan Nazir, Omur Besbinar, Cansu Gurcan, Neus Lozano, Luis M. Arellano, Süleyman Yalcin, Oguzhan Panatli, Dogantan Celik, Damla Alkaya, Aydan Agan, Laura Fusco, Serap Suzuk Yildiz, Lucia Gemma Delogu, Kamil Can Akcali, Kostas Kostarelos, Açelya Yilmazer

**Affiliations:** ^1^ Stem Cell Institute Ankara University Balgat Ankara 06520 Turkey; ^2^ Ministry of Health General Directorate of Public Health Microbiology References Laboratory Sihhiye Ankara 06430 Turkey; ^3^ Department of Chemistry Ankara University Tandogan Ankara 06100 Turkey; ^4^ Department of Biomedical Engineering Ankara University Golbasi Ankara 06830 Turkey; ^5^ Catalan Institute of Nanoscience and Nanotechnology (ICN2) UAB Campus Bellaterra Barcelona 08193 Spain; ^6^ Department of Biomedical Sciences University of Padua Padua 35122 Italy; ^7^ Department of Biophysics Faculty of Medicine Ankara University Sihhiye Ankara 06230 Turkey; ^8^ Nanomedicine Lab National Graphene Institute and Faculty of Biology Medicine & Health The University of Manchester AV Hill Building Manchester M13 9PT United Kingdom

**Keywords:** antiviral therapeutics, COVID‐19, in silico, in vitro, viral mutations

## Abstract

Nanotechnology can offer a number of options against coronavirus disease 2019 (COVID‐19) acting both extracellularly and intracellularly to the host cells. Here, the aim is to explore graphene oxide (GO), the most studied 2D nanomaterial in biomedical applications, as a nanoscale platform for interaction with SARS‐CoV‐2. Molecular docking analyses of GO sheets on interaction with three different structures: SARS‐CoV‐2 viral spike (open state – 6VYB or closed state – 6VXX), ACE2 (1R42), and the ACE2‐bound spike complex (6M0J) are performed. GO shows high affinity for the surface of all three structures (6M0J, 6VYB and 6VXX). When binding affinities and involved bonding types are compared, GO interacts more strongly with the spike or ACE2, compared to 6M0J. Infection experiments using infectious viral particles from four different clades as classified by Global Initiative on Sharing all Influenza Data (GISAID), are performed for validation purposes. Thin, biological‐grade GO nanoscale (few hundred nanometers in lateral dimension) sheets are able to significantly reduce copies for three different viral clades. This data has demonstrated that GO sheets have the capacity to interact with SARS‐CoV‐2 surface components and disrupt infectivity even in the presence of any mutations on the viral spike. GO nanosheets are proposed to be further explored as a nanoscale platform for development of antiviral strategies against COVID‐19.

## Graphical abstract

Detailed in silico and in vitro tools are applied to show that graphene oxide is able to interact effectively with the SARS‐CoV‐2 surface proteins and receptors leading to an infection inhibitory action. Four viral clades are used to determine inhibition of viral infection following mutations in the viral proteins.

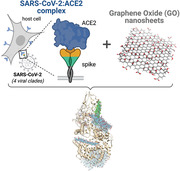

## 1 Introduction

SARS‐CoV‐2 has spread all over the world, over 192 countries with more than 146 million confirmed cases and 3.1 million deaths.^[^ ^1^ ^]^ Even though there are numerous preclinical and clinical studies ongoing, unfortunately, there is currently no specific preventive or curative treatment for coronavirus disease 2019 (COVID‐19).^[^ ^2^, ^3^ ^]^ As discussed in various reports, nanotechnology and nanomaterials could provide solutions for the fight against COVID‐19 in different ways, including: i) detection of viral particles; ii) protection of human exposure from viral particles via personal protection equipment; iii) inactivation of viral particles by capture onto surfaces or different environments; and iv) inactivation of viral particles through molecular surface interactions with novel therapeutic drug molecules (e.g., small molecules or antibodies) and vaccines.^[^ ^4^ ^]^

Since the early phases of the pandemic, computational models have been used to screen for drug molecules previously approved for other pathologies and disease conditions to be potentially repurposed against COVID‐19.^[^ ^5^, ^6^ ^]^ Via these approaches, drugs such as remdesivir or toremifene have been reported to show high binding affinities against SARS‐CoV‐2 proteins, including the spike protein or main protease.^[^ ^7^, ^8^, ^9^, ^10^ ^]^ Following these in silico and further pre‐clinical evaluations, these drugs have been moved toward clinical trials, and remdesivir has recently been approved by the Food and Drug Administration for the treatment of COVID‐19 requiring hospitalization.^[^ ^11^ ^]^ During these computational simulations, different approaches can be used: Docking analysis is one of those, used to illuminate the interaction processes of molecules that are spatially interacting to each other. The most critical information to be obtained from this process is to identify the ligand(s) that bind to protein epitopes most efficiently and determine the energy values of such binding interactions. In light of this information, one can anticipate to reveal new target proteins and the ways in which potential drug molecules could interact with them.^[^ ^12^, ^13^ ^]^ Molecular dynamics simulations (MDS) have mainly been used for conformation analysis of proteins. Conformational change processes take place in a short period of time (nanosecond to microsecond) and are impossible to follow experimentally. MDS enables us to visualize such experimentally challenging events. From such analysis, the aim has been to elucidate the role that amino acids play in ligand interactions and which amino acids provide structural stability to the protein.^[^ ^14^, ^15^ ^]^

Several viral mechanisms can be shown as targets including the blockade of structural proteins responsible for entry into human cells or inhibition of important viral enzymes responsible for genome replication or viral assembly.^[^ ^2^, ^3^ ^]^ Receptor binding domains (RBD) of the viral spike play a critical role in SARS‐CoV‐2 and ACE2 fusion, so this is a very relevant target complex for viral inhibition.^[^ ^16^ ^]^ As described above, various therapeutic strategies have been suggested to inhibit this interaction. Among some of the clinically tested drugs, chloroquine/hydroxychloroquine, arbidol, ribavirin, pensiclovir, favipiravir, nafamostat, nitazoxanide, and camostat mesylate have been shown to decrease virus–host cell binding or cellular internalization or release.^[^ ^17^, ^18^, ^19^, ^20^, ^21^, ^22^ ^]^ Delineating the mechanisms of virus and cell interactions and viral life cycle, is not only important for the advancement of antiviral therapeutics, but also for the development of protective technologies from these viruses. For this reason, antiviral agents which show higher binding affinity and neutralization activity of viral particles have been included in the fabrication of personal protective equipment, including face masks. Copper, zinc, and polyethylenimine (PEI) are among such agents with antiviral activity that have been previously reported to increase inactivation efficiency in masks by improved viral entrapment without any toxicity to the user.^[^ ^23^, ^24^, ^25^, ^26^, ^27^ ^]^

Following the discovery of graphene, different classes of 2D materials including transition‐metal dichalcogenides (e.g., MoS_2_ and WSe_2_), transition metal carbides (MXenes, e.g., Ti_3_C_2_), hexagonal boron nitride (hBN), and graphitic carbon nitride (g‐C_3_N_4_) have been developed.^[^ ^28^, ^29^, ^30^, ^31^ ^]^ Due to their unique physicochemical properties including optical and electronic properties, along with their diverse chemical composition and physicochemical properties including their good biocompatibility, these nanomaterials have attracted attention in various disciplines, including biomedical sciences.^[^ ^32^, ^33^ ^]^ Currently, applications such as anticancer therapeutics, multimodal bioimaging, cancer theranostics, biosensing, tissue engineering, and antimicrobial coatings have been proposed and studied. Out of these 2D materials, graphene oxide (GO) is the most extensively explored in biomedicine. Various groups have shown how GO sheets interact with biological systems and fully characterize their toxicological, biodegradability, and tissue distribution profiles by detailed in vitro and in vivo experiments.^[^ ^34^, ^35^, ^36^, ^37^, ^38^ ^]^

The current study aims to offer a combined in silico and in vitro analysis to interrogate whether GO sheets can be considered as a nanomaterial platform able to interact effectively with specific SARS‐CoV‐2 surface proteins and receptors leading to an inhibitory action. Molecular docking experiments have been performed to understand how GO interacts with the viral spike, the ACE2 cell receptor, and the spike‐ACE2 complex and identify the binding parameters that govern these interactions. Finally, a proof‐of‐concept study was performed in vitro (using Vero E6 cell cultures) to experimentally evaluate the effect of GO on the inhibition of wild‐type SARS‐CoV‐2 infectivity. Four different infectious viral clades, as classified by the Global Initiative on Sharing All Influenza Data (GISAID) platform, have been used in order to better understand the inhibition of viral infection in the presence of mutations in viral proteins.

## 2 Results

**Figure 1:**
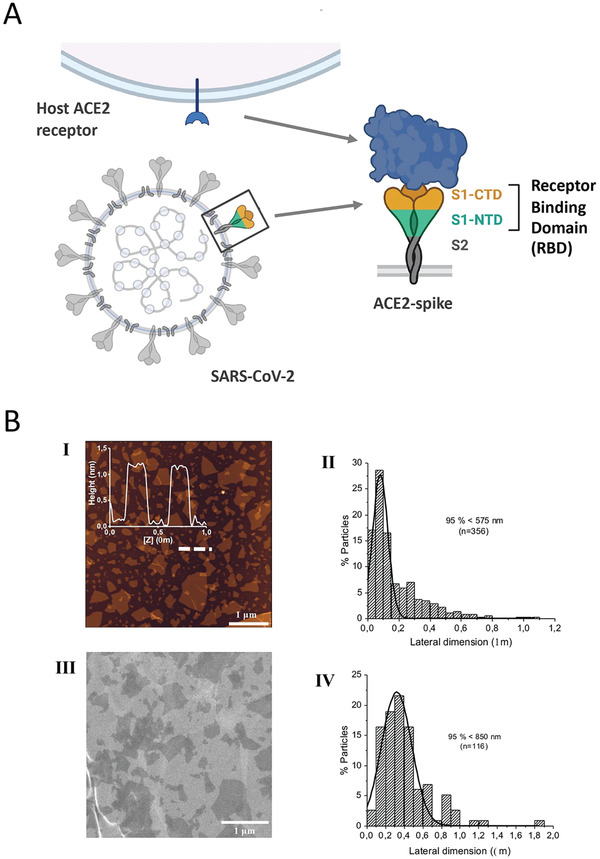
A) Schematic representation of viral spike and ACE2. SARS‐CoV‐2 binds to host cell receptor ACE2 through spike protein. Receptor binding domain (RBD) plays an important role during this interaction. Created with BioRender.com. B) Characterisation of GO material. I) Height AFM image (dimension: 5×5; scale bar: 1 µm) with insert of height cross‐section profile along the indicated region in the height AFM image; II) corresponding lateral dimension distribution analysis (number of analyzed flakes: 356); III) SEM micrograph (scale bar: 1 µm); and IV) corresponding lateral dimension distribution (*n* = 116 analyzed flakes).

**Figure 2:**
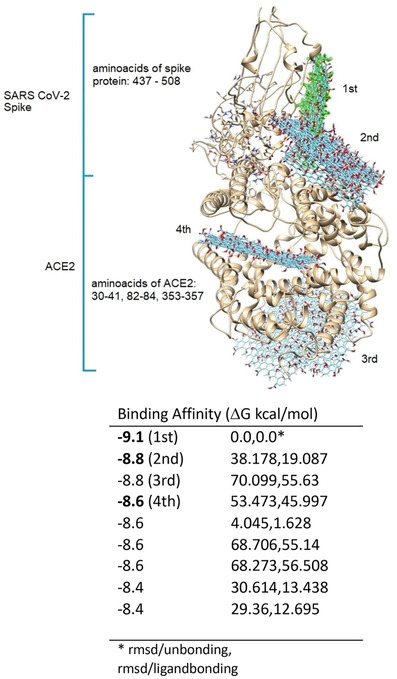
The top nine docking results of GO and 6M0J. The binding regions of GO are numbered considering the binding affinity values. GO colored with green represents the highest binding affinity. Binding affinities of these identified regions are given on the right.

**Figure 3:**
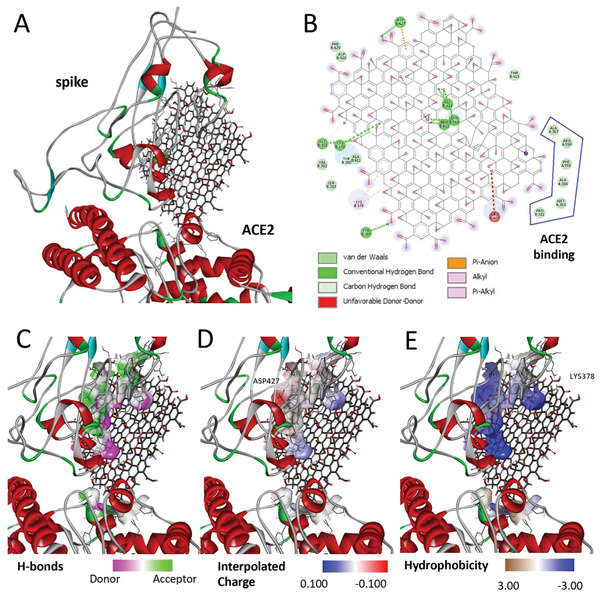
Detailed investigation of GO and 6M0J docking analysis. A) GO‐6M0J docking result, binding affinity −9.1 kcal mol^−1^; B) 2D map of 6M0J amino acids bonding interactions with GO. Spike residues: TYR369, LYS378, CYS379, TYR380, GLY381, VAL382, SER383, ARG408, ALA411, PRO412, GLY413, GLN414, THR415, ASP427, ASP428, PHE429. ACE2 residues: PRO321, MET383, ALA384, ALA383, PHE555, ARG559; C) H‐bonding: pink shows donors and green shows acceptors; D) Charge interaction with ASP427; E) Hydrophobicity (alkyl and pi‐alkyl) interactions, LYS378.

It has been shown that 6M0J is the ACE2‐bound form of SARS‐CoV‐2 spike protein. According to the UniProt (The Universal Protein Resource) database (https://www.uniprot.org/uniprot/P0DTC2), the 437–508 receptor‐binding motif area of the 319–541 amino acid receptor binding domain (RBD) of spike proteins binds to the human‐ACE2 protein (**Figure** **1**A). The amino acids 30–41, 82–84, and 353–357 of ACE2 proteins are involved in this interaction (https://www.uniprot.org/uniprot/Q9BYF1). Examining the affinity of GO for the 6M0J could allow us to examine its effect on both the spike and ACE2 binding. The docking calculation results of GO and 6M0J are shown in **Figure** **2**. Considering the docking results of GO with the 6M0J, GO showed affinity in 4 regions of the spike‐ACE2 pair between values −9.1 kcal mol^−1^ to −8.4 kcal mol^−1^. However, three of these binding conditions (1st, 2nd, and 4th) are particularly important since they cover the entire SARS‐CoV‐2 infection process (spike, ACE2 and interface). When the highest binding affinity of GO‐Spike‐ACE2 docking (−9.1 kcal mol^−1^) was examined in detail, GO showed strong affinity against amino acids of the spike‐RBD via 2 hydrophobic, 12 hydrogen, and 1 charge interaction with ASP427, a total of 15 bonds, and as well as 6 van der Waals forces which all explained the strong interaction with the ACE2 (**Figure** **3**). On closer examination, (Table S2, Supporting Information) GO could act as a multiple H‐donor and H‐acceptor with strong H‐bonds ranging from 1.74 Å to 2.93 Å. Moreover, electrostatic‐pi–anion interaction of 427 ASP spike residue with GO is important for both increasing the binding strength and affecting the load density in the close chemical environment. In addition, GO formed van der Waals bonds with the following amino acid residues of ACE2: PRO321, MET383, ALA384, PHE55, ARG559, and ALA387. The 2nd and 4th positions of the GO:6M0J docking results are also shown in Figure S2, Table S3, and Figure S3, Table S4, respectively). Overall, it could be concluded that GO strongly bound on the surface of 6M0J.

**Figure 4:**
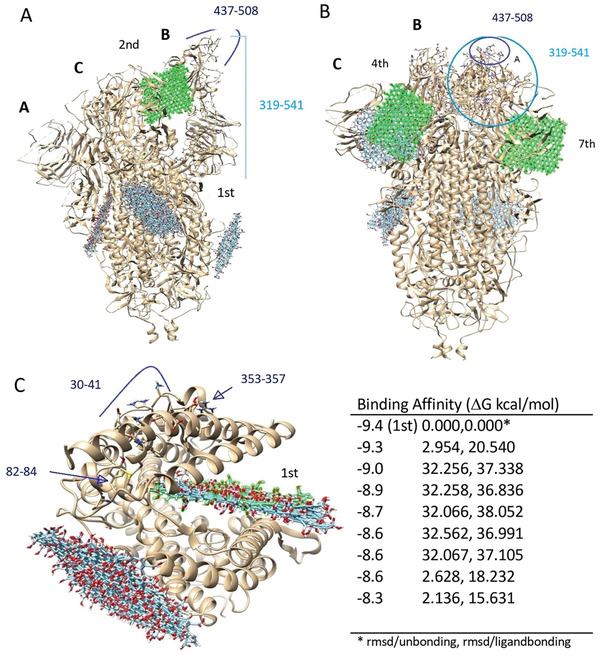
Docking of GO against open/closed state of Spike and IR42. A) GO with open state of spike (6VYB), 2nd affinity (highlighted in green) value corresponds to −10.4 kcal mol^−1^; B) GO with closed state of spike (6VXX), 4th and 7th affinity values correspond to −8.7 and −8.4 kcal mol^−1^, respectively. C) The top nine docking results of GO and 1R42 are given on the right. The binding regions of GO are numbered considering the binding affinity values. GO colored with green represents the highest binding affinity.

The 3‐stranded glycoproteins 6VYB and 6VXX of SARS‐CoV‐2 are those binding to ACE2 and correspond to open and closed states of the SARS‐CoV‐2 spike, respectively. The interaction of GO with these proteins is shown in **Figure** **4**A,B and the affinity values are given in Table S4, Supporting Information. As seen in Figure 4A,B, GO has the potential to interact with each region of open and closed sate glycoproteins. The affinity values of these interactions ranged between −10.5, −9.4 and −9.0, −8.3 kcal mol^−1^, respectively (Table S5, Supporting Information). These high affinity values indicate that the bond strength should also be high. In addition, when the binding positions of GO in glycoproteins were examined, this indicated also an effective interaction, especially in relation to the RBD regions of the glycoproteins. GO acted as a strong hydrogen donor and acceptor (Tables S6 and S7; Figure S4–S6, Supporting Information) on interaction with both glycoproteins in the range of 2.214–3.326 Å. In addition, GO has been shown to produce electrostatic interactions with both proteins (6VYB: A: LSY417; 6VXX: B: LYS462, C: ARG273, B: GLU465, C: ASP88) (Figures S4d and S5d, Supporting Information), while the chemical surface character of GO can change the charge load density.

During a SARS‐CoV‐2 infection event, blocking of the interaction between the virus and host cells could be achieved by directly acting on the viral particles, the host cell receptors or the viral particles bound to the host receptor. To interrogate such possible interactions, we performed docking simulations between GO and ACE. Figure 4C shows the docking results of GO with 1R42. When Figures 2 and 4C were evaluated comparatively, it was seen that the binding positions of GO and spike on the ACE2 do not overlap. However, when binding affinity values are taken into account for these docking analyses, the difference between them was 0.8 kcal mol^−1^, indicating that GO binds more strongly to ACE2. This difference between binding strengths comes from the excess and diversity of the bonds formed between the GO and the ACE2 (Figures S3b and S6b, Supporting Information). When the bonding interactions were compared (Tables S4 and S8, Supporting Information), 7 hydrogen bonds and 2 hydrophobic interactions are obtained between GO and ACE2 binding (6M0J), while 12 hydrogen bonds, 2 hydrophobic, and 1 electrostatic interaction are calculated for ACE2. Therefore, it can be concluded that GO bound more strongly to the ACE2.

Based on the in silico investigations above, GO had been shown to have a strong affinity toward both the viral spike and the ACE2. In order to interrogate the possible implications of such interactions experimentally, we performed in vitro tests using infectious, wild‐type SARS‐CoV‐2 virions. The biological‐grade GO material used in the studies has been developed for biomedical applications by our laboratories and are currently extensively explored in the context of various labs and projects. The characterization data of the specific batch of GO materials used in these studies are described in Figure 1B; and Figure S1, Supporting Information, in agreement with our previous reports.^[^ ^39^, ^40^, ^41^, ^42^, ^43^ ^]^ The GO sheets used were thin (1–3 carbon layers) and of a few hundred nanometers in lateral dimension (100–400nm), with proven biocompatibility and purity.

**Figure 5:**
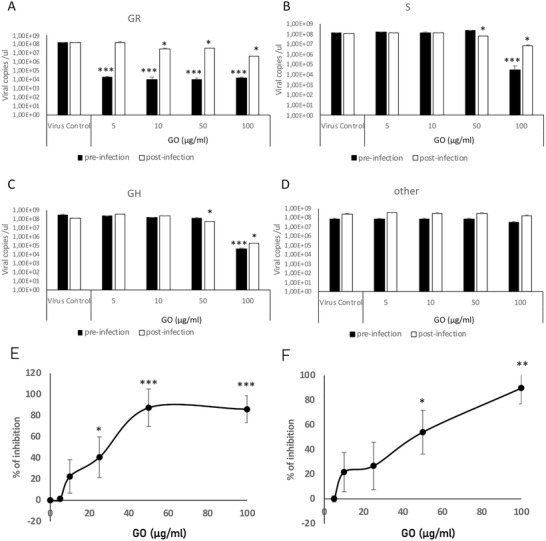
In vitro evaluation of GO mediated viral inhibition. Vero E6 cells were treated with GO (5, 10, 50, and 100 µg mL^−1^) and SARS‐CoV‐2 viral particles (MOI 0.1) according to a pre‐infection or post‐infection protocol. Four different viral genotypes were used which belong to A) GR, B) S, C) GH, and D) other clades according to GISAID. After 5 days, cell culture supernatants were used to quantify viral copy numbers by qRT‐PCR. Vero E6 cells were treated with GO (5, 10, 50, and 100 µg mL^−1^) and SARS‐CoV‐2 viral particles (MOI 0.1) from clade GR according to E) pre‐infection or F) post‐infection protocol. % of inhibition was plotted following plaque assay. * *p* < 0.05, ***p < 0.001 compared to virus control.

Prior to testing antiviral activity, the possible cytotoxic impact of GO exposure to Vero E6 cells has been evaluated. As can be seen from Figure S7, Supporting Information, material did not show any significant toxicity up to 100 µg mL^−1^ in vitro in Vero E6 cells. Four different viral genotypes that have been previously deposited in GISAID platform, all corresponding to a different clade, were used for viral infection experiments. During in vitro experimentation, two different protocols were followed: a pre‐infection and a post‐infection protocol. In the pre‐infection protocol, Vero E6 cells were first treated with the GO at different concentrations, and after 2 h viral particles at MOI of 0.01 were added into the cell culture media. During the post‐infection protocol, cells were first incubated with the infectious virions and GO at different concentrations were added later onto the infected cell culture. Clade GR showed the highest and significant inhibition of infection in response to GO treatment (**Figure** **5**A), reaching nearly a four‐log reduction in viral copy number following the pre‐infection protocol. On the other hand, S and GR showed significant inhibition only at the highest GO dose of 100 µg mL^−1^ (Figure 5B and 5C). The clade named as “other” was the least responsive to GO‐mediated inhibition, although still a significant reduction of viral copy number was achieved at 100 µg mL^−1^ GO concentration (Figure 5D). Plaque‐forming assays were also performed in order to further validate the observed antiviral activity of GO against SARS‐CoV‐2 from the GR clade. As can be seen in Figure 5E, treating the Vero E6 cells with GO prior to viral infection with the GR clade resulted in an inhibition profile from which the median inhibitory concentration (IC50) was found to be around 30 µg mL^−1^. Whereas for the post‐infection protocol, a higher IC50 value (45 µg mL^−1^) was obtained (Figure 5F). Arbidol which has been shown to be an effective drug in disrupting viral uptake in Vero E6 cells^[^ ^18^ ^]^ has been also used as a “benchmark” therapeutic agent in our study. When the Vero E6 cells were pretreated with Arbidol at different dilutions, inhibition of viral infection could be observed both via qRT‐PCR and plaque assays (Figure S8, Supporting Infomration). This data supports that in both assays, which have been also extensively used in literature,^[^ ^20^, ^21^, ^44^ ^]^ the GO antiviral activity could also be demonstrated. When the pre‐infection and post‐infection protocols were compared, it could be seen that the inhibition level of viral infection was higher during the pre‐infection protocol, as evident by both qRT‐PCR and plaque assays. This agreed with the in silico analysis, since GO had been shown to have higher affinity toward the spike or ACE2 (a situation mimicked by pre‐infection) compared to the spike‐bound ACE2 (a situation mimicked by post‐infection).

**Figure 6:**
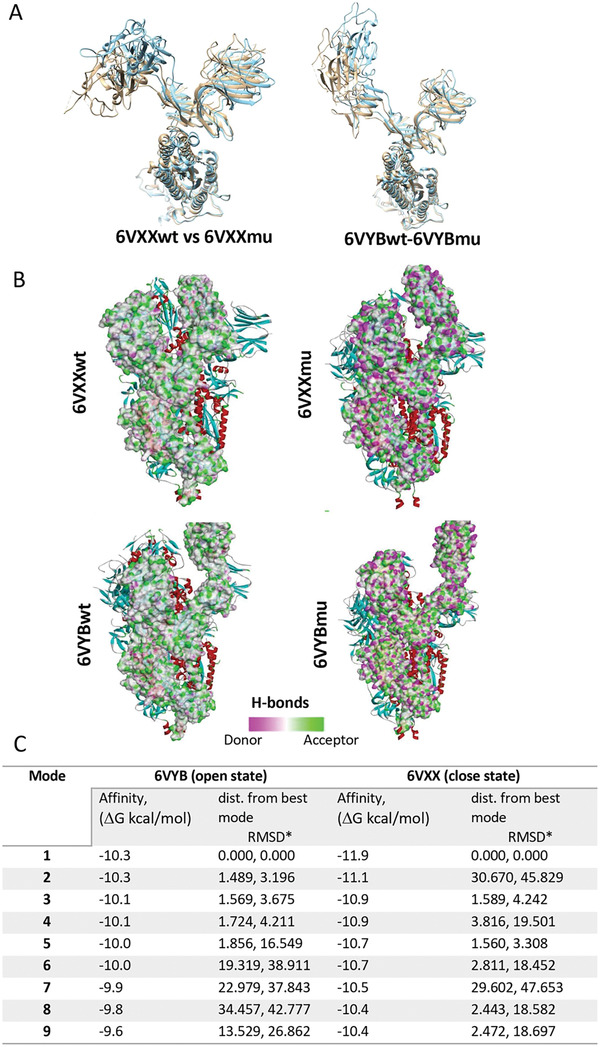
The effect of the D614G mutation on the interaction between GO and spike. A) Comparison of wild type and mutation structures of 6VXXwt‐6VXXmu and 6VYBwt‐6VYBmu (colors – tawny‐brown: wt; cyan: mutant) shows that the structural change occurred at the RBD. B) The D614G mutation effect on B wire of 6VXX and 6VYB are obtained. C) Molecular docking of GO against the mutated form of 6VXX and 6VYB was performed and binding affinities (ΔG kcal mol^−1^) of 6VYBmu and 6VXXmu were calculated.

Finally, we went back to the in silico platform and have delineated the effect of mutations found in the four different viral genotypes used in the in vitro study. Molecular docking was applied to better explain the experimental results obtained in vitro. The mutation profile of all genotypes can be found in Table S9, Supporting Information. GO is expected to mainly interact with the viral surface proteins and/or host cell surface receptors. For this reason, while considering the mutations on the viral genome, we mainly focused on the mutations of the spike protein. Both the GR and GH clades have a mutation at the spike structure, which is D614G. The GR clade showed more sensitivity against the GO compared to the GH clade, pointing out the importance of mutations found in the downstream viral proteins. However, all of these mutations are on the non‐structural genes in the GH clade and their impact on overall viral infectivity has not yet been studied enough. The S clade, on the other hand, has a different spike mutation known as G1251V which is not within the receptor binding domain of the viral spike, and therefore is not expected to play an important role in the spike recognition. The clade represented as “other” didn't show any mutation on the viral spike, representing a native form of the spike protein. We have performed molecular docking of GO on interaction with the mutated spike D614G, which is one of the mostly reported mutations causing increased infectivity of SARS‐CoV‐2 due to its effect on the receptor binding domain of the spike protein. To examine the effect of the D614G mutation on the spike protein, first, a structural prediction was made by replacing the 614th amino acid Asp in the B chain of 6VXX and 6VYB with Gly. After structural prediction, the structural comparison – RMSD values between wild type and mutant structures were calculated to be 7.869 Å and 7.919 Å for 6VXXwt‐6VXXmu and 6VYBwt‐6VYBmu, respectively. The structural changes between the wild‐type and the mutant viruses occurred in the RBD regions of the spike protein (**Figure** **6**A). This structural change altered the donor–acceptor properties of the amino acid chain (Figure 6B) and consequently led to an increased binding strength and interaction between the GO sheets and the spike proteins (Figure 6C).

## 3 Discussion

Nanotechnology offers a number of possibilities for antiviral activity, both outside and inside the host cells. Several nanotechnology‐based platforms have already been successful in preclinical studies to counter a variety of human viral pathogens such as HIV, human papilloma virus, herpes simplex, and respiratory viruses.^[^ ^45^, ^46^, ^47^ ^]^ Moreover, nanoscale systems can potentially increase the effectiveness of drugs and other bioactive molecules by reducing the required effective dose, therefore dramatically improve the therapeutically effective drug toxicity thresholds. In the literature, there are numerous reports of antiviral drug delivery platforms suitable for different viral diseases and targets. Such platforms include liposomes, emulsions, de÷rimers, micelles, solid‐lipid hydrogel based nanocarriers, polymeric nanoparticle, carbon‐based and 2D materials.^[^ ^48^, ^49^, ^50^ ^]^

The field of nanotechnology has recently caught the attention of computational analysis in order to help scientists offer better understanding of nanomaterial interactions with biological systems.^[^ ^4^ ^]^ Based on the data generated by such *in silico* analyses, nanotechnology‐based therapeutic approaches could be improved.^[^ ^51^, ^52^, ^53^ ^]^ Among 2D materials, GO is the most studied one using such computational models to depict interactions with various biological moieties, mainly proteins.^[^ ^54^, ^55^, ^56^, ^57^, ^58^, ^59^, ^60^, ^61^ ^]^ For example, Baweja et al. reported that GO and reduced GO (rGO) were able to inhibit the α‐helix to β‐sheet transition of amyloid beta (Aβ) peptide, which has been implicated in the pathogenesis of Alzheimer's disease.^[^ ^55^ ^]^ In another study, Putri el al. studied via computational analysis the thermally responsive behavior of a polymer‐GO complex in the design of a sensor with an “on/off” switch upon binding to a cancer cell marker at its lower critical solution temperature.^[^ ^61^ ^]^ *In silico* approaches are becoming valuable tools to better understand the therapeutic potential of nanomaterial‐drug conjugates, including 2D materials.

Considering the urgent need to offer as many options as possible in managing the SARS‐CoV‐2 pandemic, such computational methods are especially important to guide the rational design of new nanoscale systems on interaction with the critical SARS‐CoV‐2 viral components. In our study, GO was shown to have affinity towards the spike protein, ACE2 receptors and spike‐ACE2 complex. However, when the binding affinities and types of bonds were compared, GO was found to interfere more strongly to the viral spike (6VYB or 6VYB) and the ACE2 (1R42) before binding to the virus ligand, compared to 6M0J. Based on this *in silico* observation, when cells were exposed to GO prior to viral treatment in the context of a pre‐infection protocol closer to a preventive clinical senario, pronounced viral inhibition was observed compared to a post‐infection protocol in vitro. In combination, *in silico* and in vitro analyses in this study emphasized the importance of the correlation between computational and experimental methodologies in evaluating the antiviral activity of nanoscale platforms suspended in physiologically relevant aqueous solutions.

2D nanomaterials, due to their extremely large surface area, can be superior carriers for antiviral drug delivery purposes compared to other materials with different structural conformations and dimensions. 2D nanomaterials indeed have been explored in various preclinical studies for the delivery of cytotoxic agents, such as MTX, DOX or 5‐FU (among others) to cancer cells for chemotherapeutic applications.^[^ ^62^, ^63^, ^64^ ^]^ Although much attention has been placed on cancer therapy, there are studies suggesting that these materials are also promising candidates for anti‐microbial therapies. Experimental studies have shown that the interaction between graphene‐related 2D materials and bacteria, viruses and fungi could lead to strong anti‐bacterial and antiviral activities.^[^ ^65^ ^]^ For example graphene oxide (GO) derivatives have been shown to compete with the cell surface receptor heparan sulfate in binding herpes simplex virus type‐1 (HSV‐1).^[^ ^66^ ^]^ Another study reported the broad‐spectrum antiviral activity of GO against pseudorabies virus (PRV, a DNA virus) and porcine epidemic diarrhea virus (PEDV, an RNA virus). According to these reports, GO significantly suppressed the infection of PRV and PEDV at non‐cytotoxic concentrations.^[^ ^67^ ^]^ Deokar et al. also showed the design and synthesis of sulfonated magnetic nanoparticles functionalized with reduced graphene oxide (SMRGO) to capture and photothermally destroy HSV‐1.^[^ ^68^ ^]^ In a more recent study by Donskyi et al., a series of graphene derivatives with defined polyglycerol sulfate and fatty amine functionalities have been synthesized and their interactions with HSV‐1 were investigated. When 2D sheets were functionalized with C_6_‐ and C_9_‐alkyl chains, they showed efficient inhibition of HSV‐1 without any significant toxicity in VeroE6 cells, suggesting that antiviral agents against HSV‐1 can be obtained by controlled and stepwise functionalization of graphene sheets.^[^ ^69^ ^]^

Recently, several groups started reporting the antiviral activities of different nanomaterials against the novel SARS‐CoV‐2 virus. Nanoparticles containing silver, aluminum nitride or copper have been suggested to inhibit SARS‐CoV‐2 infectivity.^[^ ^70^, ^71^, ^72^, ^73^ ^]^ In another approach, Zhang et al. constructed cellular nanosponges which display cellular receptors of the virus on the surface, to demonstrate that these nanosponges were able to neutralize viral particles resulting in inhibition of infection.^[^ ^74^ ^]^ In contrast, studies evaluating the antiviral activity of 2D materials are very limited. In one of two studies to do so, Raval et al. reported a simple, initial computational analysis showing interaction between graphene and the receptor‐binding domain of spike complexed with its receptor ACE2. The molecular simulation data using pristine multi‐layer graphene reported interactions with SARS‐CoV‐2 proteins, but no experimental work was offered to validate the computational observations.^[^ ^75^ ^]^ However, inhibition of viral infection can happen not only at the spike – ACE2 complex, but also at the spike or ACE2. As can be seen in our study, comparing docking analysis at different protein domains is crucial to better evaluate the inhibitory effect of nanomaterials. In the context of graphene or GO incorporated in personal protective equipment (PPE), De Maio et al. recently reported that GO could reduce SARS‐CoV‐2 infectivity in vitro.^[^ ^76^ ^]^ However, the GO concentration range selected is excessive and not realistic for the purposes of antiviral therapeutics. Furthermore, given that there are different viral clades spreading among patient populations, it was important to evaluate the effectiveness of graphene or GO functionalized PPEs against multiple infectious viral genotypes.

In our present study, it is shown that GO can lead to reduced SARS‐CoV‐2 infectivity in 3 out of 4 infectious viral clades tested. Differences in viral inhibition among viral clades could be attributed to the mutations found in the viral genotypes. When GO was docked against one of the mutated regions, enhanced binding affinity was observed. For example, D614G mutation in the viral spike, which has also been reported to cause higher infectivity among human populations, was found to cause a structural change at 6VXX.^[^ ^77^, ^78^, ^79^, ^80^ ^]^ Based on this knowledge and the experimental data obtained in vitro, when GO was computationally docked against one of these mutated spike regions, enhanced binding affinity was observed. We postulate that further interference of all viral genotype mutations that may be encountered in human populations can be achieved via appropriate engineering the GO material properties via surface functionalization. In addition to the D614G mutation, it has been recently reported in GISAID platform that the other most common receptor binding mutations S477N (part of large Melbourne outbreak from clade GR and some Central European clade GH clusters), N439K (the long lasting UK outbreak with clade G and the European spillover), N501Y (the new UK variant VUI‐202012/01 in clade GR and a recent clade GH outbreak in South Africa) and Y453F (the mink adaptation) as well as combinations of these mutations with deletions alter the surface of spike protein. These changes will certainly affect its affinity for host receptors, as well as the antiviral nanomaterials being developed. Therefore, our findings actually show the importance of considering different viral genotypes/mutations that will arise also in the future, in order to better understand the effect of nanomaterials tested against SARS‐CoV‐2. Overall, our observations suggest that GO can be considered a promising candidate to be used as an antiviral platform nanomaterial in the design of either PPE able to capture and retain viral particles^[^ ^76^ ^]^ for disease prevention, or as an antiviral drug delivery system for therapeutic purposes.

## 4 Experimental Section

##### Molecular Docking

The crystal structures of 6M0J^a^, 1R42^b^, 6VXX^c^, and 6VYB^c^ were obtained from the Research Collaboratory for Structural Bioinformatics Protein Data Bank (RSCB PDB).^[^ ^81^, ^82^, ^83^ ^]^ These protein domains have been identified as: 6M0J: SARS‐CoV‐2 spike receptor‐binding domain bound with ACE2, 1R42: native human Angiotensin Converting Enzyme (ACE2), 6VXX: SARS‐CoV‐2 spike glycoprotein (closed state) and 6VYB: spike ectodomain structure (open state). Prior preparation of protein domains (screening, removing waters, small molecules, and more), docking analysis, and image processing after analysis were all performed by Chimera version 1.14 and Discovery Studio 2020, which have been well‐established in literature.^[^ ^84^, ^85^ ^]^ The free‐standing GO surface (with functional groups on both sides of the layer) is optimized using modified AMBER force field and MD simulations. The split in the oxygen groups of GO is about: Epoxy oxygen 15% Hydroxy oxygen 10% Carboxy (edge groups) 5% (% calculated with respect to the total carbon atoms on the graphene layer). GO and protein models prepared in PDB format were converted to PDBQT format with the addition of Gasteiger partial charges via the AutoDock program version 4.2,^[^ ^86^ ^]^ and grid box mapping parameters were also determined (Table S1, Supporting Information). In order to observe the affinity of GO against the whole protein structure, grid box parameters were adjusted to cover the whole protein. Finally, molecular docking analysis of GO and protein prepared in PDBQT format were performed using AutoDock‐Vina,^[^ ^87^ ^]^ which is a sensitive and high‐performance docking software. As a result of the calculations, nine results with the highest binding affinity were evaluated. For mutant analysis, the program Robetta was used for B chain structural prediction of 6VYB and 6VXX proteins (https://robetta.bakerlab.org).

##### Material (Graphene Oxide) Synthesis and Characterisation

*Synthesis of Biological‐Grade GO*: Biological‐grade GO was synthesized from graphite powder (Sigma–Aldrich, UK) by the modified Hummers’ method as previously described.^[^ ^88^ ^]^ Depyrogenation of all the glassware involved in the synthesis and the use of non‐pyrogenic material was used to obtain endotoxin‐free GO material suitable for biological applications.^[^ ^38^ ^]^ Briefly, 18.4 mL of sulfuric acid was added to the mixture of the reaction containing 0.8 g of graphite and 0.4 g of sodium nitrate. Then, 2.4 g of potassium permanganate was added very slowly. After 30 min, 37.5 mL of water was transferred slowly and the reaction was kept at 98 °C for 30 min. Afterward, 112.5 mL of water was added and also 12.5 mL of 30% hydrogen peroxide to stop the reaction. The consequent GO purification was performed by centrifugation steps until the pH of the supernatant was neutral. GO material was obtained after 5 min sonication and then purified by centrifugation.^[^ ^39^ ^]^

*Atomic Force Microscopy*: An Asylum MFP‐3D atomic force microscope (Oxford instruments) operating in standard air‐tapping mode and equipped with silicon probes (Ted Pella) with a resonance frequency of 300 kHZ and a nominal force of 40 N m^−1^ was used to characterize the surface. Samples were prepared by drop casting of 20 µL of GO suspension (100 µg mL^−1^) onto a freshly cleaved mica surface (Ted Pella) previously covered with 20 µL of poly‐L‐lysine 0.01% solution (Sigma‐Aldrich), subsequently washed with water, and then dried overnight at room temperature. Images were processed using Gwyddion software (http://gwyddion.net, version 2.56). Distribution analysis of 5 µm x 5 µm height AFM images to obtain the lateral dimensions was performed using ImageJ software (https://imagej.nih.gov).

*Scanning Electron Microscopy*: SEM images were recorded on a Magellan 400L field emission scanning electron microscope (Oxford instruments) at the ICN2 Electron Microscopy Unit, which was equipped with an Everhart‐Thornley as secondary electrons detector and using an acceleration voltage of 20 kV and beam current of 0.1 nA. In each sample, 20 µL of GO material (100 µg mL^−1^) were deposited on an Ultrathin C on Lacey C grid; any excess of material was removed and dried overnight at room temperature. The lateral dimension distribution was obtained by measuring the flakes using ImageJ software.

*Raman Spectroscopy*: Raman spectra were acquired with a confocal Raman microscope (Witec) at room temperature coupled to 632 nm laser excitation and using a grating of 600 g nm^−1^. Single Raman spectra were collected on several spots after irradiation with a power of 1 mW for 10 s and using a 50x objective to focus on the sample. Samples were prepared by drop casting of 20 µL onto glass coverslip and then, dried overnight. The data were collected and analyzed with Origin software. The intensity ratio ID/IG was obtaining by taking the peak intensities without baseline correction.

*Zeta Potential Measurements*: Zeta potential (ζ) was measured by a Zeta‐sizer Nano ZS (Malvern instruments) equipped with disposable capillary cells at the ICN2 Molecular Spectroscopy and Optical Microscopy Facility. Water dispersant settings for refractive index and viscosity, and automatic analysis were used for all GO measurements (20 µg mL^−1^). Each sample was measured three times at room temperature.

*UV–Vis Spectroscopy*: Absorbance was evaluated by using a Nanodrop 2000c spectrophotometer (Thermo Scientific) at room temperature using a Hellma QS Quartz micro cuvette. GO samples were prepared in water in a concentration range of 2.5–20 µg mL^−1^.

*X‐Ray Photoemission Spectroscopy*: XPS data was obtained using a Phoibos 150 (SPECS, GmbH) electron spectrometer equipped with a hemispherical analyzer, operating under ultrahigh‐vacuum conditions, and with an Al Kα (hν = 1486.74 eV) X‐ray source, at the ICN2 Photoemission Spectroscopy Facility. Charge effects on the samples were removed by taking the C1s line from adventitious carbon at 284.6 eV. Samples were prepared by deposition of 20 µg of GO material onto 5×5 silicon wafers (Ted Pella) and dried overnight. In order to estimate the photoelectron peak intensities, CasaXPS software (http://www.casaxps.com) was used.

##### Cell Culture

African green monkey kidney Vero E6 cell line was purchased from ATCC and maintained in DMEM media containing 10% FBS and 1% antibiotics. Four local SARS‐CoV‐2 isolates (hCoV‐19/Turkey/HSGM‐302/2020 (Clade GR), hCoV‐19/Turkey/HSGM‐439/2020 (Clade other), hCoV‐19/Turkey/HSGM‐1049/2020 (Clade GH), and hCoV‐19/Turkey/HSGM‐1027/2020 (Clade S) with GISAID accession numbers of EPI_ISL_437313|2020‐03‐27, EPI_ISL_437307|2020‐03‐25, EPI_ISL_437309|2020‐03‐26 and EPI_ISL_437317|2020‐03‐27, respectively) was used in this study. Viruses were propagated in Vero E6 cells by using DMEM media containing 2% FBS and 1% antibiotics. All virus‐related experiments were performed at Biosafety Level 3 laboratories.

##### Viral Infection

Vero E6 cells were seeded onto 96‐well plates at confluency. Cells were either incubated with GO (5, 10, 50, 100 µg mL^−1^) or SARS‐CoV‐2 (MOI 0.1) in a pre‐infection or post‐infection protocol. In pre‐infection protocol, cells were first treated with 100 µL media containing GO and after 2 h, viral particles were added onto the culture media. In a post‐infection protocol, cells were first infected with SARS‐CoV‐2 (100 µL) and following 2 h of incubation, GO was added into the wells. After the final treatment, plates were incubated at 5% CO_2_ at 37 °C. Plates were monitored for cytopathic activity for 5 days.

##### qRT‐PCR Analysis

At the end of day 5, cell culture supernatants were collected. Total RNA was isolated via MPLC total nucleic acid isolation kit (Roche) using automated MagNA Pure LC Instrument (Roche). One‐step qRT‐PCR was performed using the transcriptor one‐step RT‐PCR Kit (Roche) by using 5 µL of samples per each 20 µL reaction volume. In order to calculate viral copies per µL, a standard curve was constructed by using five different standards of known copy numbers according to the viral N gene.

##### Plaque Assay

Vero E6 cells were seeded onto 48‐well plates at confluency. Cells were either incubated with GO (5, 10, 50, 100 µg mL^−1^) or SARS‐CoV‐2 (MOI 0.1) in a pre‐infection or post‐infection protocol. After 1h, carboxymethyl cellulose (CMC) overlay medium (300 µL) was added into each well. At the end of 4 days, plates were fixed with 10% formaldehyde and stained with crystal violet. Plaques were counted for each condition and plaque forming units were calculated. Based on pfu values, % of inhibition was calculated for each treatment group compared to the virus control group.

##### Statistical Analysis

All values were expressed as mean ±ST.D. Comparison between groups was performed by one‐way ANOVA, followed by a Tukey's post hoc multiple comparisons by the statistics program GraphPad. At least three independent replicated were analyzed.

## Conflict of Interest

The authors declare no conflict of interest.

## Supporting information

Supporting Information

## Data Availability

The data that support the findings of this study are available from the corresponding author upon request.
