# Supplementary material for: Graphene Oxide Nanosheets Interact and Interfere with SARS‐CoV‐2 Surface Proteins and Cell Receptors to Inhibit Infectivity
Source: Small. 2021 May 14;17(25):2101483. doi: 10.1002/smll.202101483 (PMC8236978; doi:10.1002/smll.202101483)
Supplement: Supplementary file 1 — Supporting Information [file SMLL-17-2101483-s001.pdf]

© 2021 Wiley-VCH GmbH

NANO MICRO small

Supporting Information

for *Small,* DOI: 10.1002/smll.202101483

Graphene Oxide Nanosheets Interact and Interfere with SARS-CoV-2 Surface Proteins and Cell Receptors to Inhibit Infectivity

Mehmet Altay Unal, Fatma Bayrakdar, Hasan Nazir, Omur Besbinar, Cansu Gurcan, Neus Lozano, Luis M. Arellano, Süleyman Yalcin, Oguzhan Panatli, Dogantan Celik, Damla Alkaya, Aydan Agan, Laura Fusco, Serap Suzuk Yildiz, Lucia Gemma Delogu, Kamil Can Akcali, Kostas Kostarelos, and Açelya Yilmazer**

Supplementary Information

Graphene oxide nanosheets interact and interfere with SARS-CoV-2 surface proteins and cell receptors to inhibit infectivity

Mehmet Altay Unal[#], Fatma Bayrakdar[#], Hasan Nazir, Omur Besbinar, Cansu Gurcan, Neus Lozano, Luis M. Arellano, Süleyman Yalcin, Oguzhan Panatli, Dogantan Celik, Damla Alkaya, Aydan Agan, Laura Fusco, Serap Suzuk Yildiz, Lucia Gemma Delogu, Kamil Can Akcali, Kostas Kostarelos*, Açelya Yilmazer*

Table S1. Grid box mapping parameters of proteins used in Autodock-Vina*.

	6M0J	1R42	6VXX	6VYB
Center (Å)				
x	-24.7020	58.0255	222.6740	218.406
y	18.5065	59.8315	209.4370	212.116
z	-7.2405	26.2695	195.4800	201.544
Box dimension (Å)				
x	55.354	73.341	110.888	107.874
y	67.701	61.091	121.915	113.728
z	110.897	58.989	155.792	168.452

*other parameters: energy_range = 3, exhaustiveness = 8, num_modes = 9

Table S2. Details of bonding interactions of GO and 6M0J (Figure 2b, 1st position).

Donor-Acceptor	Distance, Å	Category	Types*	Angle, DHA*	Angle, HAY(Z,E)*
GLY381:HN - GO:O108	2.930	H-bond	HB	131.012	124.431
GLN414:HE21 - GO:O301	2.414	H-bond	HB	120.328	96.332
GLN414:HE21 - GO:O468	2.383	H-bond	HB	103.382	112.085
GLN414:HE22 - GO:O468	2.587	H-bond	HB	91.048	106.153
GO:H100 - CYS379:O	2.424	H-bond	HB	106.036	140.75
GO:H289 - GLY413:O	1.686	H-bond	HB	147.166	137.477
GO:H440 - ASP427:OD2	2.637	H-bond	HB	154.080	126.233
GO:H506 - TYR369:OH	2.283	H-bond	HB	115.752	108.316
GO:H491 - PRO412:O	2.874	H-bond	HB	101.492	153.913
TYR380:CA -GO:O111	3.164	H-bond	CH		
GLY413:CA - GO:O296	3.458	H-bond	CH		
GO:H456 - GLY381:O	2.599	H-bond	CH		
ASP427:OD2 – GO	4.016	Electrostatic	Pi-Anion		
LYS378 – GO	4.865	Hydrophobic	Alkyl		
GO - LYS378	5.254	Hydrophobic	Pi-Alkyl		

*HB=conventional, CH=Carbon-Hydrogen; DHAY(Z,E)=donor-hydrogen-acceptor-any atom

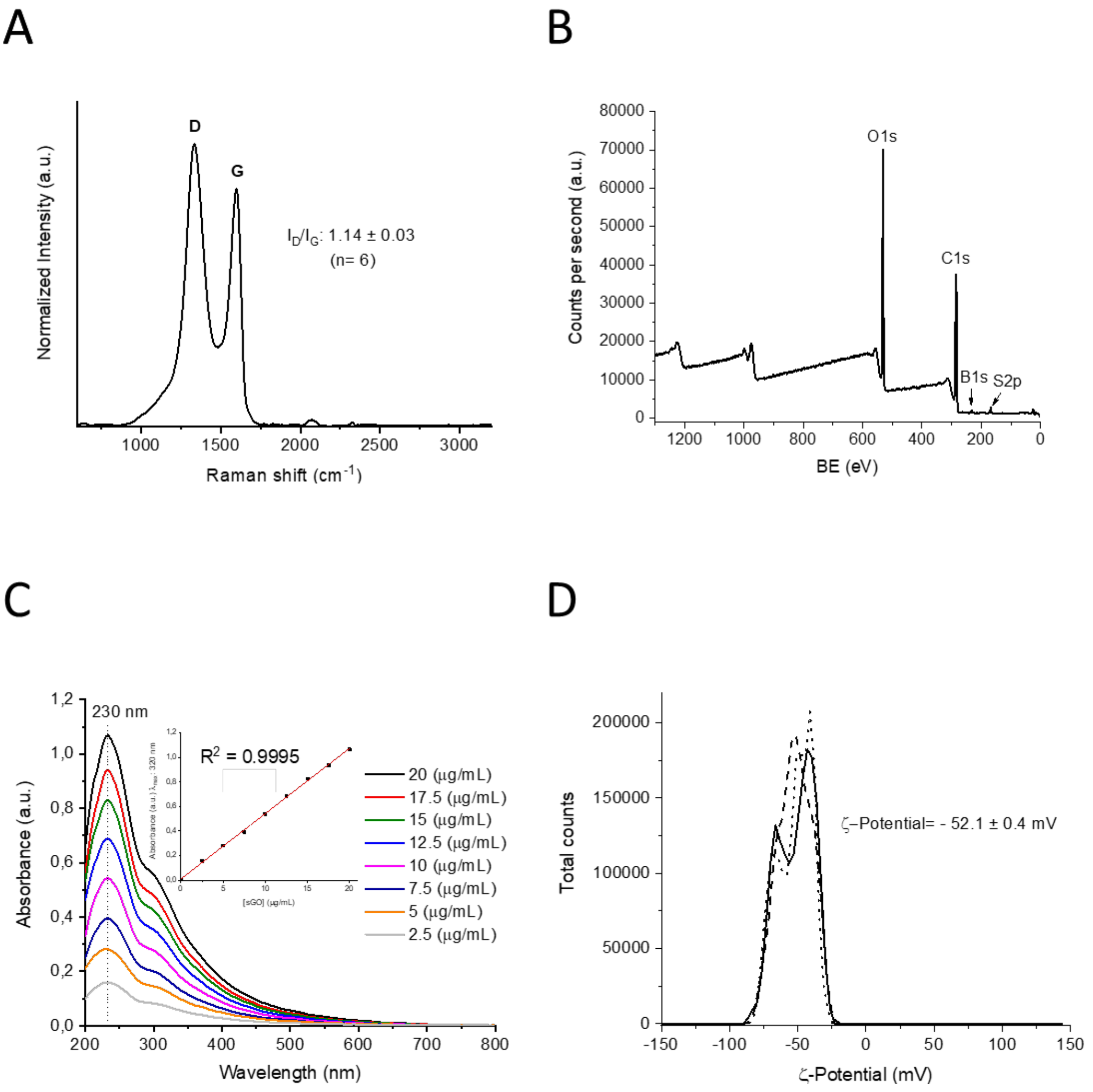

Figure S1. Additional characterization of GO material. (A) Normalized Raman spectrum as an average of 6 individual spectra. (B) XPS survey analysis. (C) UV-vis spectra ranged from 2.5 up to 20 µg/mL with calibration data at the wavelength of 230 nm. (D) ζ-Potential measurements.

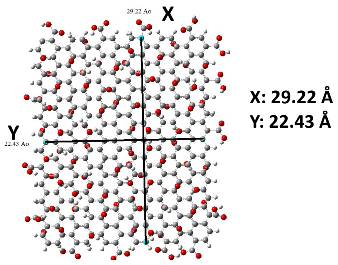

a

GO-6M0J docking results of 2nd position

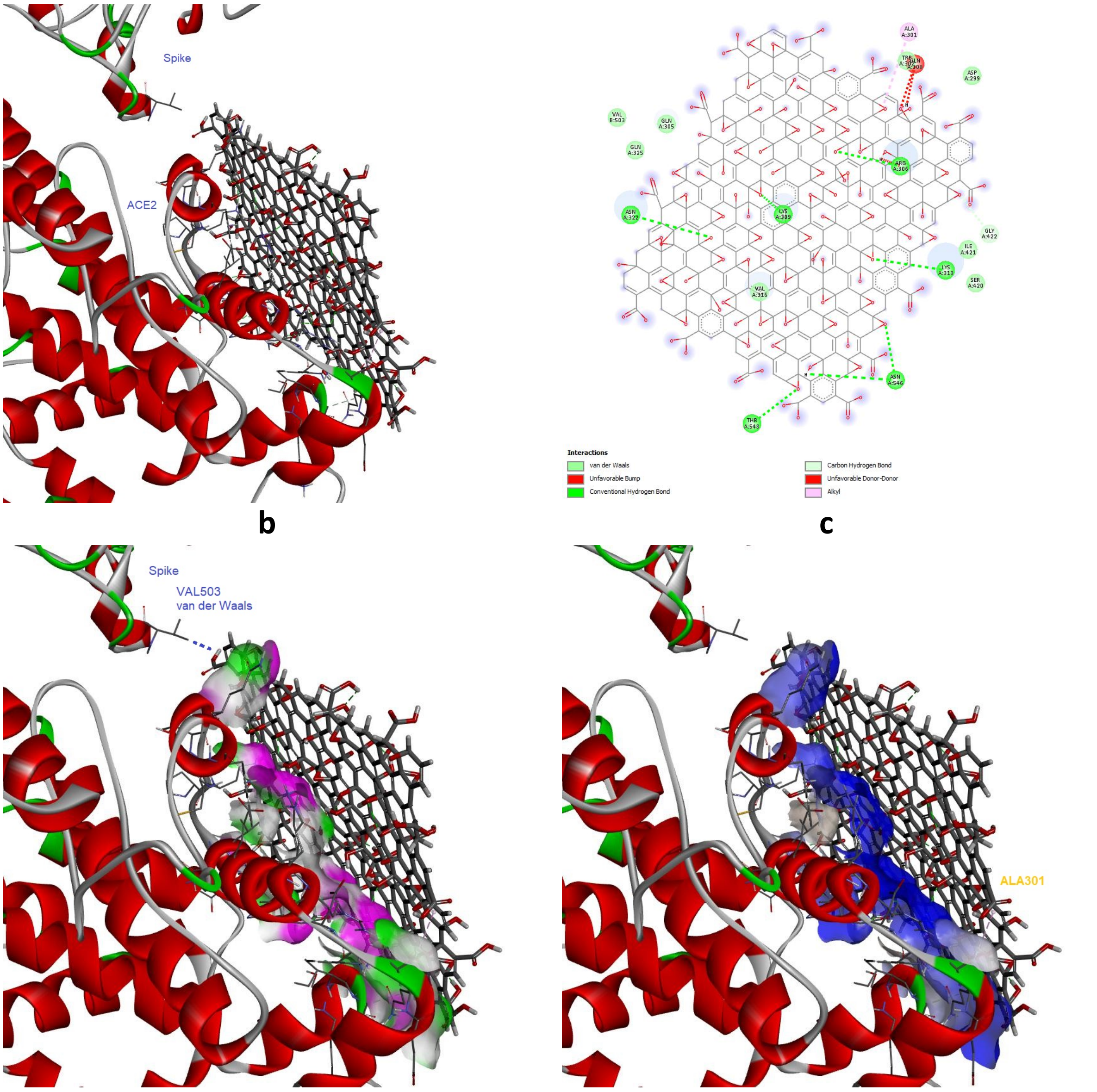

b c d e

Figure S2. (**a**) dimension of GO used in this study; (**b**) GO-6M0J docking result, binding affinity -8.8 kcal/mol; (**c**) 2D map of 6M0J amino acids bonding interactions with GO (2nd). Spike residues: VAL503 ACE2 residues: AS546, THR548, GLY422, ASN322, LYS313, LYS309, ARG306, ALA301, GLN300; (**d**) H-bonding: pink shows donors and green shows acceptors; (**e**) Hydrophobicity (alkyl) interaction, ALA301.

Table S3. Details of bonding interactions of GO and 6M0J (Figure S1b, 2nd position).

**Donor-Acceptor**	**Distance, Å**	**Category**	**Types***	**Angle, DHA***	**Angle, HAY(Z,E)***
ARG306:HH11 - GO:O471	2.412	H-bond	HB	112.74	101.987
LYS309:HZ2 - GO:O303	1.769	H-bond	HB	160.753	143.437
LYS313:HZ2 - GO:O113	3.020	H-bond	HB	105.223	116.218
LYS313:HZ3 - GO:O113	2.980	H-bond	HB	107.848	98.802
ASN322:HD21 - GO:O465	2.371	H-bond	HB	111.575	101.473
ASN546:HD21 - GO:O198	2.126	H-bond	HB	108.631	134.523
THR548:HG1 - GO:O205	3.094	H-bond	HB	120.697	104.946
GO:H193 - ASN546:O	3.049	H-bond	HB	119.992	119.231
GO:H450 - GO:O309	1.747	H-bond	HB	147.72	94.397
GLY422:CA - GO:O106	3.7485	H-bond	CB		
ALA301 - GO	4.72136	Hydrophobic	Alkyl		

*HB=conventional, CH=Carbon-Hydrogen; DHAY(Z,E)=donor-hydrogen-acceptor-any atom

GO-6M0J docking results of 4th position

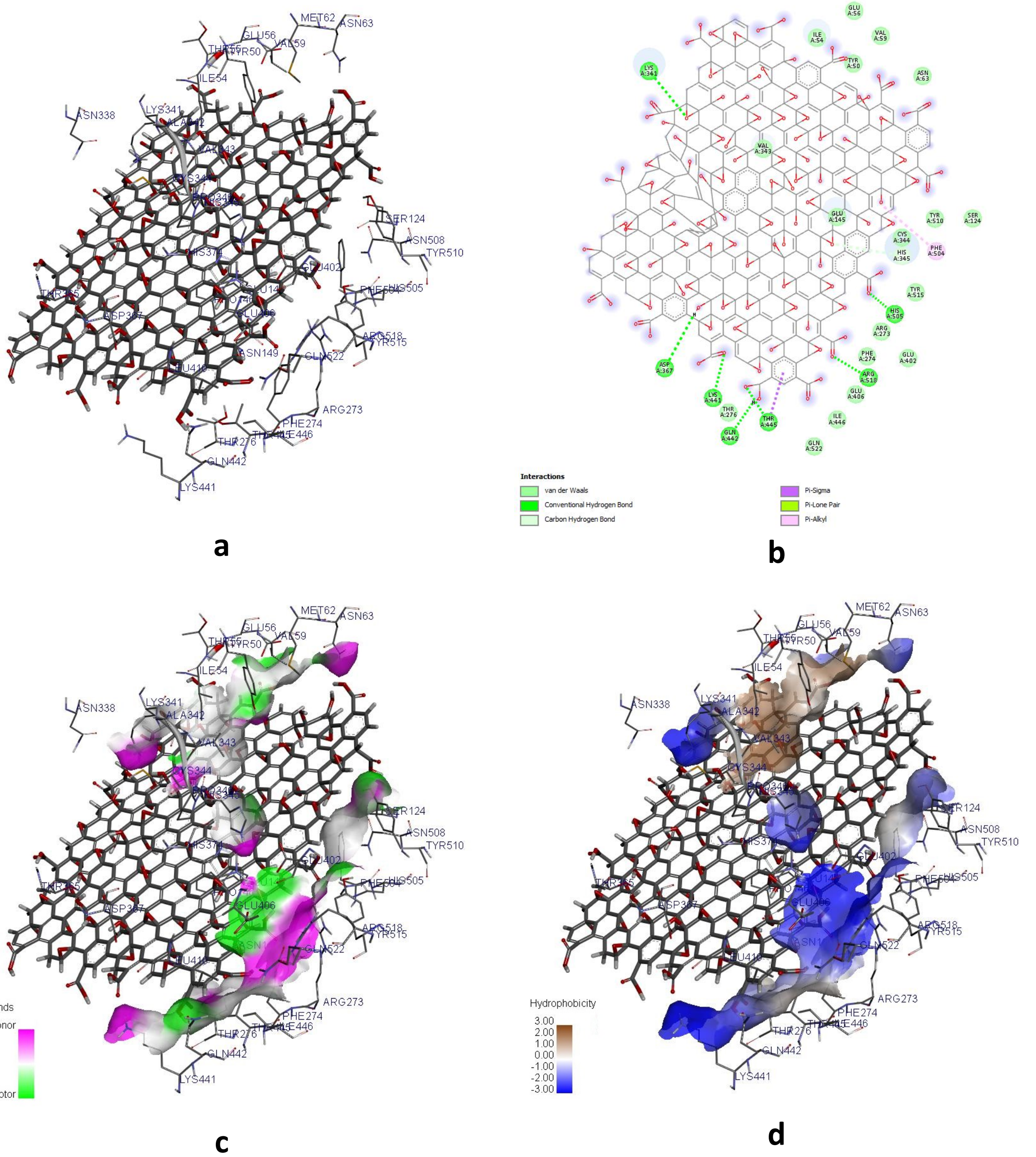

Figure S3. (**a**) GO-6M0J docking result, binding affinity -8.6 kcal/mol; (**b**) 2D map of 6M0J amino acids bonding interactions with GO (4th); (**c**) H-bonding: pink shows donors and green shows acceptors; (**d**) Hydrophobicity (Pi-Sigma and Pi-Alkyl) interaction; THR445,. PHE504.

Table S4. Details of bonding interactions of GO and 6M0J (Figure S2b, 4th position).

Donor-Acceptor	Distance, Å	Category	Types*	Angle, DHA*	Angle, HAY(Z,E)*
LYS341:HZ1 - GO:O263	2.548	H-bond	HB	126.937	98.278
LYS441:HZ1 - GO:O459	2.649	H-bond	HB	95.081	155.303
LYS441:HZ3 - GO:O459	2.311	H-bond	HB	117.661	122.436
THR445:HG1 - GO:O471	1.7915	H-bond	HB	157.091	124.165
HIS505:HE2 - GO:O455	1.769	H-bond	HB	139.265	131.891
ARG518:HH12 - GO:O487	2.468	H-bond	HB	131.386	112.018
HIS345:CD2 - GO:O97	2.531	H-bond	HB		
THR445:CG2 - GO	3.671	Hydrophobic	Pi-Sigma		
GO:O97 - A:HIS345	2.974	Other	Pi-Lone Pair		
PHE504 - GO	5.213	Hydrophobic	Pi-Alkyl		

*HB=conventional, CH=Carbon-Hydrogen; DHAY(Z,E)=donor-hydrogen-acceptor-any atom

Table S5. Binding Affinity (ΔG kcal/mol) of 6VYB and 6VXX.

Mode	6VYB (open state)		6VXX (close state)	
	Affinity, (ΔG kcal/mol)	dist. from best mode RMSD*	Affinity, (ΔG kcal/mol)	dist. from best mode RMSD*
1	-10.5	0.000, 0.000	-9.0	0.000, 0.000
2	-10.4	60.270, 73.615	-9.0	34.205, 43.802
3	-9.9	2.068, 17.335	-8.8	61.234, 80.131
4	-9.9	1.863, 17.051	-8.7	3.201, 14.741
5	-9.8	1.878, 17.271	-8.6	1.585, 4.605
6	-9.7	47.502, 56.632	-8.5	61.001, 81.189
7	-9.7	45.872, 55.156	-8.4	56.654, 72.844
8	-9.5	48.514, 57.649	-8.4	62.494, 80.007
9	-9.4	45.866, 50.776	-8.3	39.466, 51.913

* rmsd/unbonding, rmsd/ligandbonding

6VYB SARS-CoV-2 spike glycoprotein (open state)

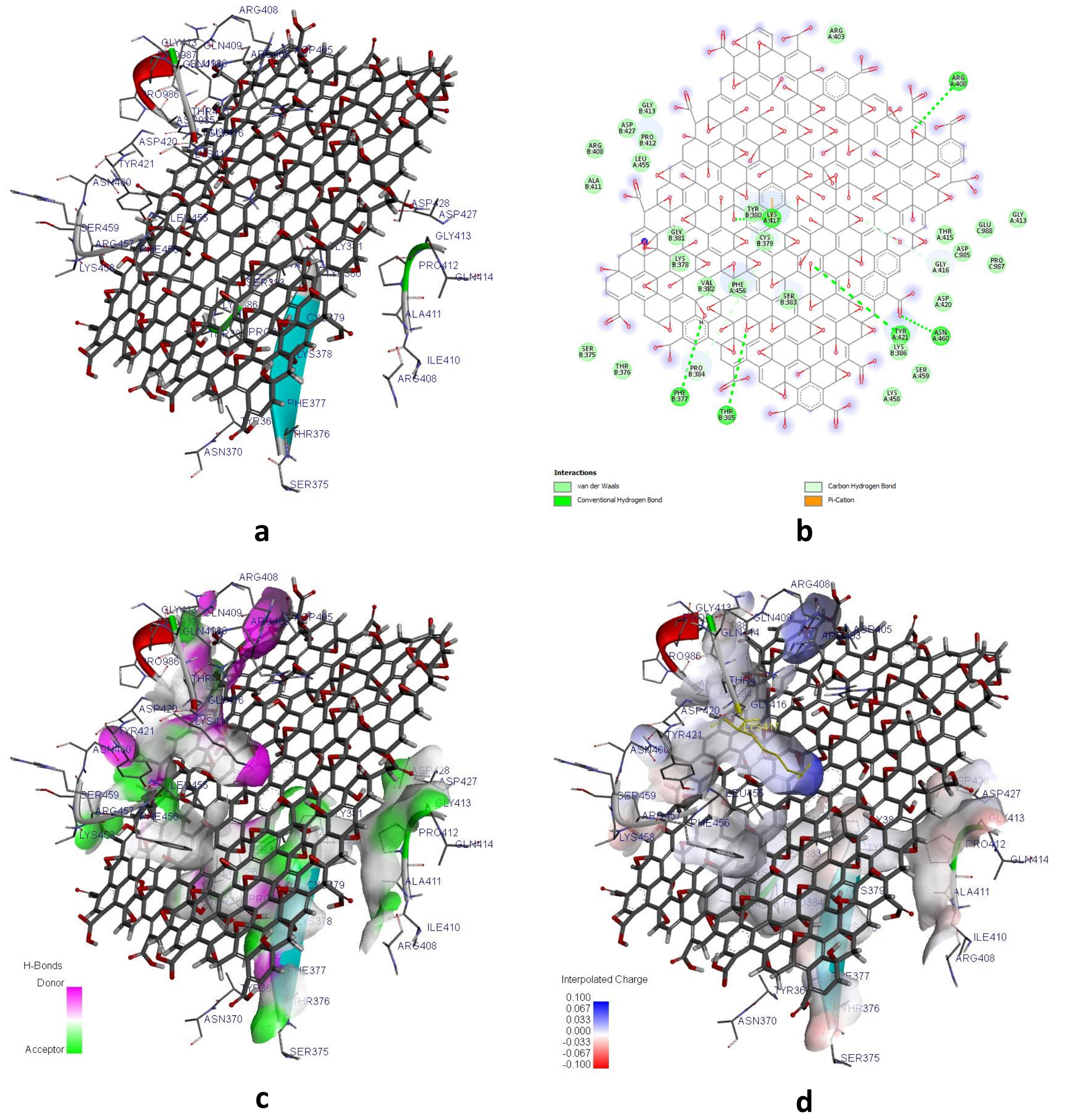

Figure S4. 2nd position of GO (**a**) binding with 6VYB; (**b**) 2D map of binding; (**c**) H-bonds; (**d**) charge interaction, A:LSY417.

Table S6. Bonding interactions of 2nd position GO with 6VYB

Donor-Acceptor	Distance, Å	Category	Types*	Angle, DHA*	Angle, HAY(Z,E)*
A:ARG408:HH21 - GO:O107	3.056	H-bond	HB	114.852	94.745
A:LYS417:HZ1 - GO:O8	2.812	H-bond	HB	134.62	105.288
A:TYR421:HH - GO:O473	3.015	H-bond	HB	123.129	91.449
A:ASN460:HD22 - GO:O445	2.645	H-bond	HB	123.379	123.395
B:THR385:HN - GO:O196	3.061	H-bond	HB	116.387	132.32
GO:H1 - B:PHE377:O	3.055	H-bond	HB	95.138	152.999
A:GLY416:CA - GO:O115	3.091	H-bond	HB		
A:LYS417:CE - GO:O109	3.281	H-bond	HB		
B:PRO384:CD - GO:O7	3.119	H-bond	HB		
A:LYS417:NZ – GO	3.545	Electrostatic	Pi-Cation		
A:LYS417:HZ1 - GO	2.653	H-bond<br>Electrostatic	Pi-Cation<br>Pi-Donor<br>HB		

*HB=conventional, CH=Carbon-Hydrogen; DHAY(Z,E)=donor-hydrogen-acceptor-any atom

6VXX SARS-CoV-2 spike glycoprotein (close state)

4th position of GO

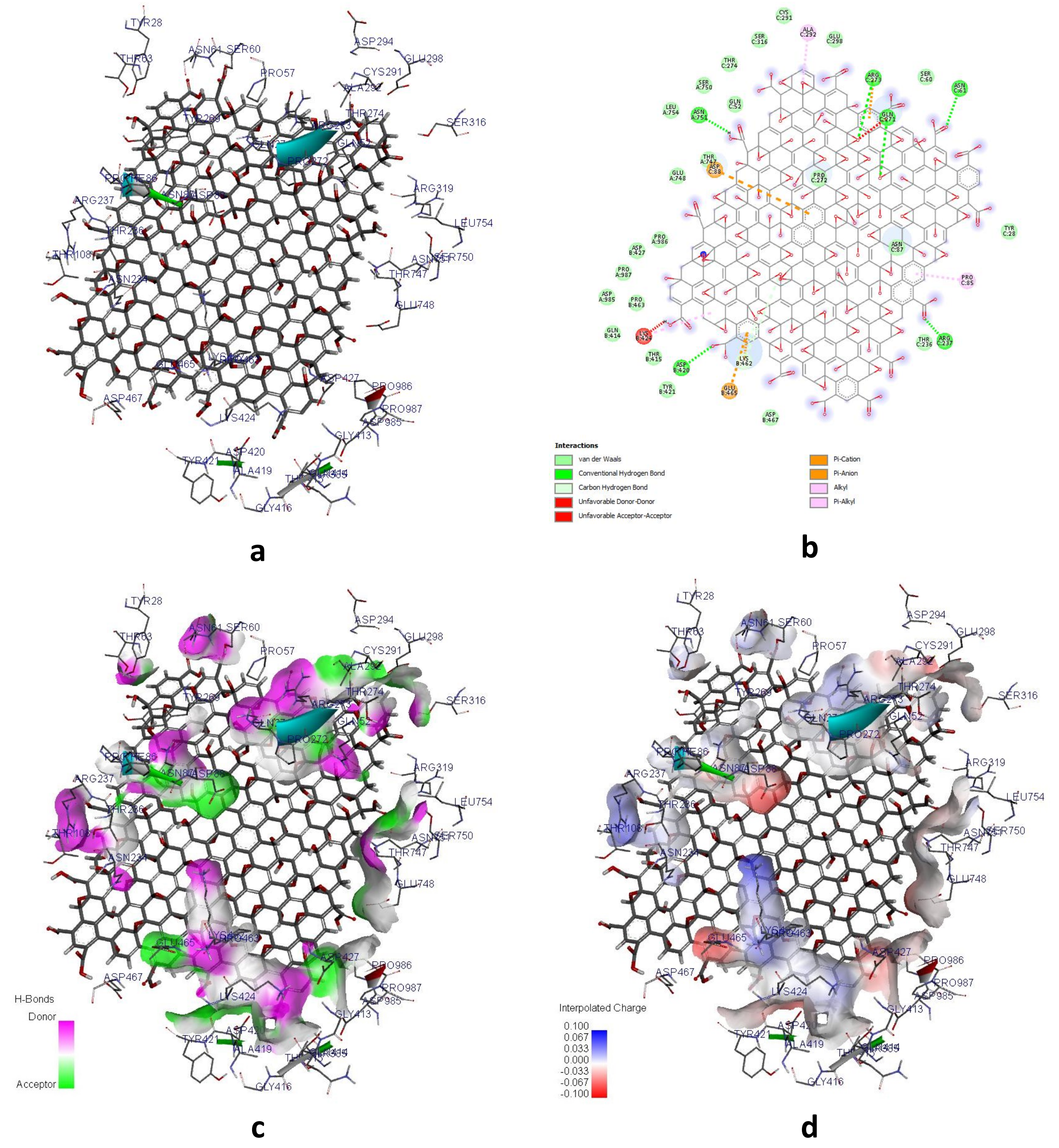

Figure S5. 4th position of GO (**a**) binding with 6VXX; (**b**) 2D map of binding; (**c**) H-bonds; (**d**) charge interaction, B:LYS462, C:ARG273, B:GLU465, C:ASP88.

Continue on next page

7th position of GO

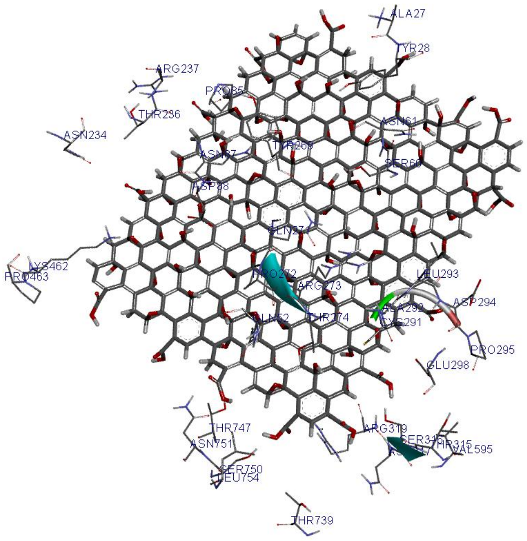

e

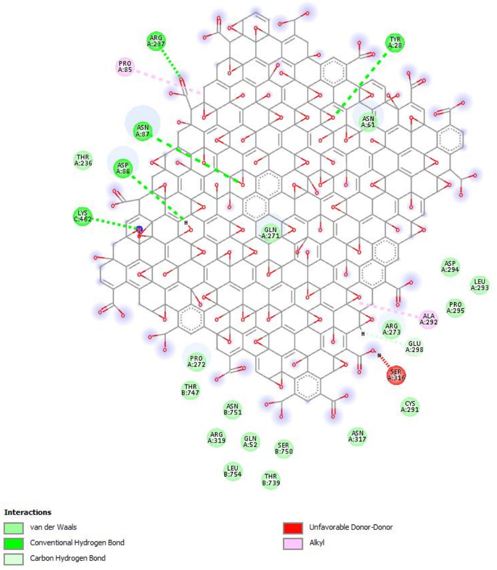

f

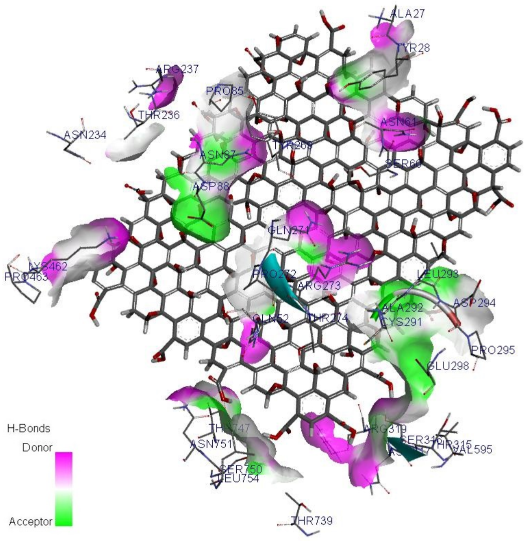

g

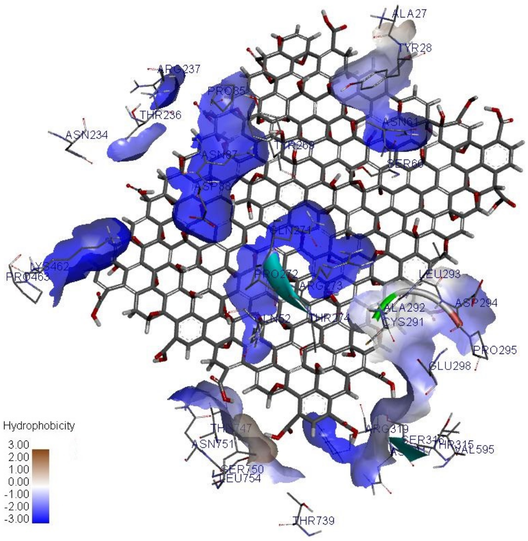

h

Figure S5 (cont). 7th position of GO (**e**) binding with 6VXX; (**f**) 2D map of binding; (**g**) H-bonds; (**h**) hydrophobic interaction, A:ALA292, A:PRO85.

Table S7. Bonding interactions of GO with 6VXX

Donor-Acceptor	Distance, Å	Category	Types*	Angle, DHA*	Angle, HAY(Z,E)*
**4th position of GO**					
C:ASN61:HN - GO:O483	2.747	H-bond	HB	145.734	170.392
C:ARG237:HE - GO:O455	2.623	H-bond	HB	136.982	158.721
C:ARG237:HH22 - GO:O455	2.145	H-bond	HB	150.705	104.761
C:GLN271:HE21 - GO:O100	2.280	H-bond	HB	99.885	103.778
C:ARG273:HE - GO:O267	2.734	H-bond	HB	125.967	142.253
C:ARG273:HH22 - GO:O267	2.438	H-bond	HB	129.505	123.98
GO:O435 - B:ASP420:O	2.659	H-bond	HB	98.720	170.474
GO:H465 - A:ASN751:OD1	2.216	H-bond	HB	153.161	140.345
B:LYS462:CE - GO:O2	2.895	H-bond	HB		
B:LYS462:CE - GO:O4	3.326	H-bond	HB		
B:LYS462:N - GO	3.643	Electrostatic	Pi-Cation		
C:ARG273:NH2 - GO	3.752	Electrostatic	Pi-Cation		
B:GLU465:OE2 - GO	4.430	Electrostatic	Pi-Anion		
C:ASP88:OD2 - GO	4.396	Electrostatic	Pi-Anion		
B:LYS424 - GO	5.444	Hydrophobic	Alkyl		
C:ALA292 - GO	3.989	Hydrophobic	Alkyl		
GO - B:LYS462	5.358	Hydrophobic	Pi-Alkyl		
GO - C:PRO85	5.004	Hydrophobic	Pi-Alkyl		
**7th position of GO**					
A:TYR28:HH - GO:O265	2.928	H-bond	HB	104.396	106.353
A:ASN87:HD22 - GO:O268	2.901	H-bond	HB	97.348	132.347
A:ARG237:HH22 - GO:O463	2.558	H-bond	HB	148.367	157.271
C:LYS462:HZ1 - GO:O1	2.045	H-bond	HB	155.517	138.928
GO:H493 - A:ASP88:OD1	2.202	H-bond	HB	111.609	102.187
GO:H379 - A:GLU298:OE2	2.512	H-bond	HB	169.006	142.973
A:ALA292 - GO	4.008	Hydrophobic	Alkyl		
GO - A:PRO85	5.185	Hydrophobic	Alkyl		

*HB=conventional, CH=Carbon-Hydrogen; DHAY(X,Z,E)=donor-hydrogen-acceptor-any atom

GO binding to ACE2

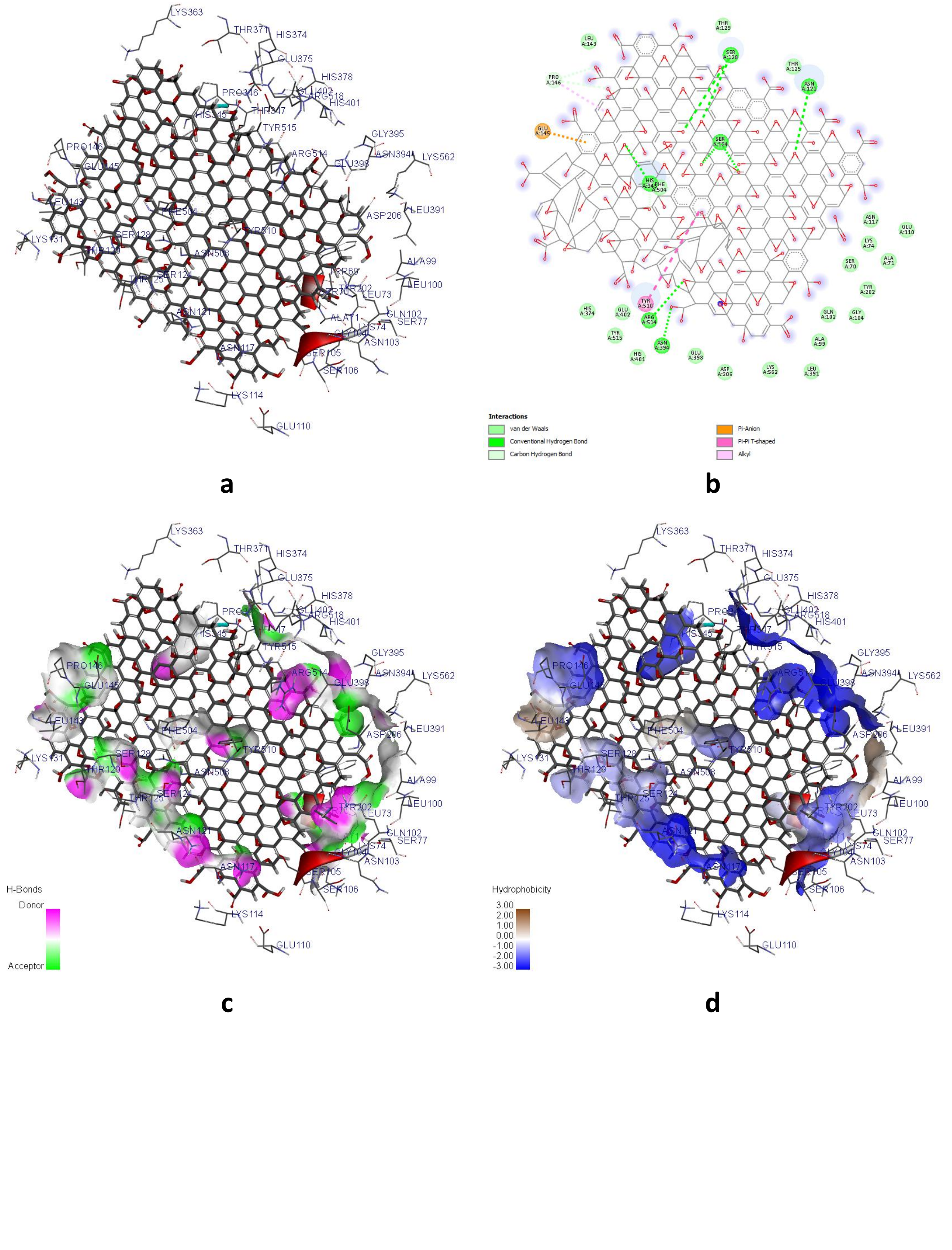

Continue on next page

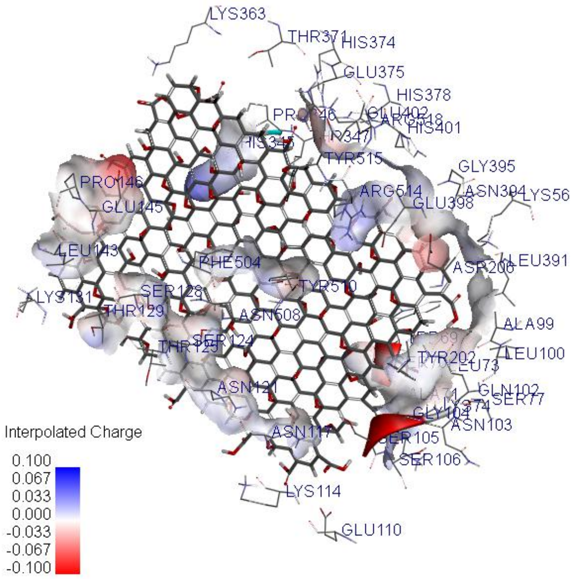

e

Figure S6. (**a**) GO-ACE2 docking result, binding affinity -9.4 kcal/mol; (**b**) 2D map of ACE2 amino acids bonding interactions with GO; (**c**) H-bonding: pink shows donors and green shows acceptors; (**d**) Hydrophobicity (Pi-Pi, T-shaped Alkyl) interaction; TYR510,. PRO146; (e) Charge, GLU145.

Table S8. Bonding interactions of GO with ACE2.

Donor-Acceptor	Distance, Å	Category	Types*	Angle, DHA*	Angle, HAY(Z,E)*
A:ASN121:HD21 - GO:O206	2.584	H-bond	HB	173.731	114.749
A:SER124:HG - GO:O209	2.570	H-bond	HB	167.883	99.58
A:SER124:HG - GO:O467	2.759	H-bond	HB	108.436	92.73
A:SER128:HG - GO:O115	2.702	H-bond	HB	175.109	114.444
A:SER128:HG - GO:O119	2.995	H-bond	HB	107.141	109.698
A:HIS345:HE2 - GO:O296	2.487	H-bond	HB	133.804	107.151
A:ASN394:HD21 - GO:O429	2.100	H-bond	HB	165.851	111.093
A:ARG514:HH11 - GO:O17	2.985	H-bond	HB	118.787	141.188
A:ARG514:HH21 - GO:O17	2.428	H-bond	HB	126.18	96.295
A:PRO146:CD - GO:O117	3.682	H-bond	HB		
A:PRO146:CD - GO:O511	3.492	H-bond	HB		
A:HIS345:CD2 - GO:O477	2.978	H-bond	HB		
A:GLU145:OE2 – GO	2.973	Electrostatic	Pi-Anion		
A:TYR510 – GO	5.821	Hydrophobic	Pi-Pi T-shaped		
A:PRO146 - GO	5.119	Hydrophobic	Alkyl		

*HB=conventional, CH=Carbon-Hydrogen; DHAY(Z,E)=donor-hydrogen-acceptor-any atom

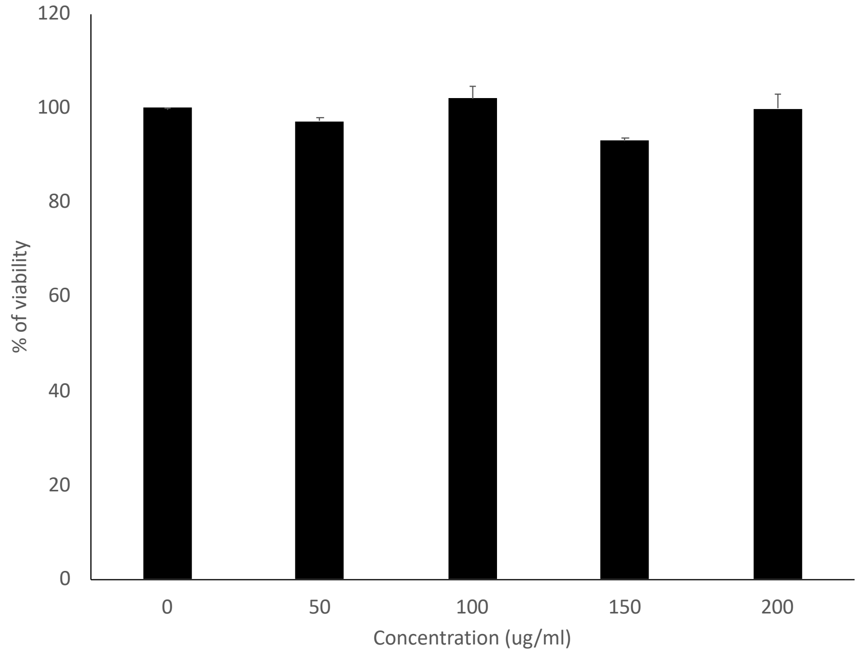

Figure S7. Toxicity of GO in Vero E6 cells. Vero E6 cells were treated with GO at different concentrations and viability of cells were assessed by modified LDH assay.

A

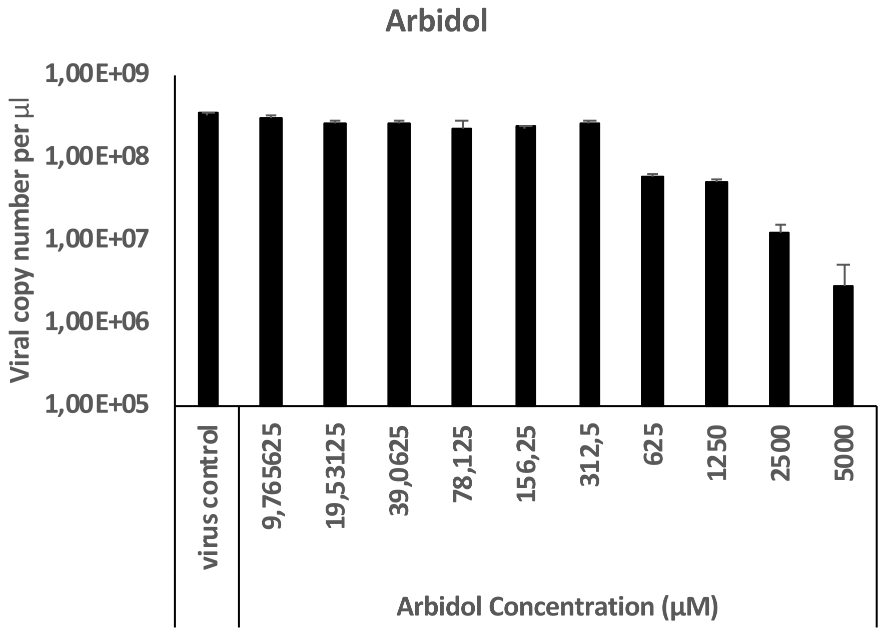

B

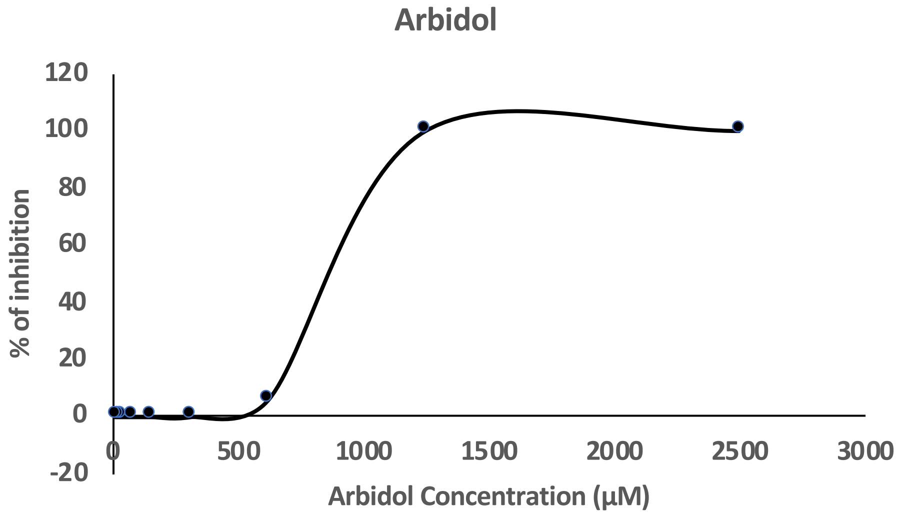

Figure S8. Anttiviral activity of the 'benchmark' drug Arbidol. Vero E6 cells were pretreated with Arbidol at different dilutions, inhibition of viral infection was assessed via A) qRT-PCR and B) plaque assay.

Table S9. Mutations of 4 viral genotypes used in this study.

Genotypes	GISAID Clade
**EPI_ISL_437313|2020-03-27**<br>Location,Mutation,Count<br>Turkey,Spike_D614G,1<br>Turkey,N_R203K,1<br>Turkey,N_G204R,1<br>Turkey,NSP12_P323L,1	GR
**hCoV-19/Turkey/HSGM-1027/2020**<br>EPI_ISL_437317|2020-03-27<br>Turkey,Spike_G1251V,1<br>Turkey,NS8_L84S,1<br>Turkey,N_S202N,1	S
**hCoV-19/Turkey/HSGM-1049/2020**<br>EPI_ISL_437309|2020-03-26<br>Turkey,Spike_D614G,1<br>Turkey,NS3_Q57H,1<br>Turkey,NSP3_P395L,1<br>Turkey,NSP2_T85I,1<br>Turkey,NSP13_T127I,1<br>Turkey,NSP12_P323L,1	GH
**hCoV-19/Turkey/HSGM-439/2020**<br>EPI_ISL_437307|2020-03-25<br>Turkey,NSP2_T170I,1<br>Turkey,NSP11_A12V,1<br>Turkey,NSP6_L37F,1<br>Turkey,NSP4_S137L,1<br>Turkey,NSP13_A4V,1<br>Turkey,NSP2_V198I,1<br>Turkey,NSP5_L50F,1<br>Turkey,NSP3_F1161L,1	Other
